# EGCG Inhibits Proliferation, Invasiveness and Tumor Growth by Up-Regulation of Adhesion Molecules, Suppression of Gelatinases Activity, and Induction of Apoptosis in Nasopharyngeal Carcinoma Cells

**DOI:** 10.3390/ijms16022530

**Published:** 2015-01-23

**Authors:** Chih-Yeu Fang, Chung-Chun Wu, Hui-Yu Hsu, Hsin-Ying Chuang, Sheng-Yen Huang, Ching-Hwa Tsai, Yao Chang, George Sai-Wah Tsao, Chi-Long Chen, Jen-Yang Chen

**Affiliations:** 1 Department of Pathology, Wan Fang Hospital, Taipei Medical University, Taipei 116, Taiwan; E-Mail: chencl@tmu.edu.tw; 2 National Institute of Cancer Research, National Health Research Institutes, Zhunan Town, Miaoli County 350, Taiwan; E-Mails: chungcwu@nhri.org.tw (C.-C.W.); kakacoco0218@gmail.com (H.-Y.H.); tw.hitomi@gmail.com (H.-Y.C.); syhuang@nhri.org.tw (S.-Y.H.); 3 Graduate Program of Biotechnology in Medicine of National Tsing Hua University and National Health Research Institutes, Hsinchu 300, Taiwan; 4 Institute of Biotechnology, Department of Life Sciences, National Tsing Hua University, Hsinchu 300, Taiwan; 5 Department of Microbiology, College of Medicine, National Taiwan University, Taipei 100, Taiwan; E-Mail: chtsai@ntu.edu.tw; 6 National Institute of Infectious Diseases and Vaccinology, National Health Research Institutes, Tainan 701, Taiwan; E-Mail: yaochang@nhri.org.tw; 7 Department of Anatomy, Li Ka Shing Faculty of Medicine, The University of Hong Kong, Hong Kong, China; E-Mail: gswtsao@hkucc.hku.hk; 8 Department of Pathology, Taipei Medical University Hospital, Taipei Medical University, Taipei 110, Taiwan

**Keywords:** EGCG, nasopharyngeal carcinoma, invasiveness, apoptosis, chemoprevention

## Abstract

(−)-Epigallocatechin-3-gallate (EGCG), a major green tea polyphenol, has been shown to inhibit the proliferation of a variety of tumor cells. Epidemiological studies have shown that drinking green tea can reduce the incidence of nasopharyngeal carcinoma (NPC), yet the underlying mechanism is not well understood. In this study, the inhibitory effect of EGCG was tested on a set of Epstein Barr virus-negative and -positive NPC cell lines. Treatment with EGCG inhibited the proliferation of NPC cells but did not affect the growth of a non-malignant nasopharyngeal cell line, NP460hTert. Moreover, EGCG treated cells had reduced migration and invasive properties. The expression of the cell adhesion molecules E-cadherin and β-catenin was found to be up-regulated by EGCG treatment, while the down-regulation of matrix metalloproteinases (MMP)-2 and MMP-9 were found to be mediated by suppression of extracellular signal-regulated kinase (ERK) phosphorylation and AP-1 and Sp1 transactivation. Spheroid formation by NPC cells in suspension was significantly inhibited by EGCG. Oral administration of EGCG was capable of suppressing tumor growth in xenografted mice bearing NPC tumors. Treatment with EGCG was found to elevate the expression of p53 and p21, and eventually led to apoptosis of NPC cells via caspase 3 activation. The nuclear translocation of NF-κB and β-catenin was also suppressed by EGCG treatment. These results indicate that EGCG can inhibit the proliferation and invasiveness, and induce apoptosis, of NPC cells, making it a promising agent for chemoprevention or adjuvant therapy of NPC.

## 1. Introduction

Nasopharyngeal carcinoma (NPC), a tumor derived from the posterior part of the nasopharynx, is a malignant neoplasm that is notorious for its local invasion and distant metastasis. The incidence of NPC is particularly high in southern China, Taiwan, Southeast Asia, and North Africa, but is very low elsewhere [1]. Genetic, environmental and microbial factors have been incriminated in the carcinogenesis of NPC [2,3]. Epstein-Barr virus (EBV) is the etiological agent of infectious mononucleosis and is implicated in the development of several human malignancies, including NPC. Clonal infection by EBV is present in most NPC biopsies and the detection of expression of several viral genes has indicated that EBV plays an etiological role in the carcinogenesis of NPC [4]. In addition to viral factors, several dietary factors have been reported to be associated with the development of NPC. Consumption of salted fish, especially during weaning, has been linked to the development of NPC [5]. Volatile nitrosamines are known to be present in salted fish and preserved foods from NPC high risk areas and are considered to be an important etiological factor of NPC [6]. Moreover, it has also been shown that various chemicals, including phorbol esters and *n*-butyrate, which are present in herbal medicines and foods commonly consumed in NPC high risk areas, can induce the EBV lytic cycle and may be involved in the tumorigenesis of NPC [7,8,9,10]. Our recent study indicated that repeated contact with these chemicals can lead to recurrent reactivation of EBV and result in alteration of cancer hallmark gene expression in NPC cells [11]. These results indicate that frequent contact with these chemicals from food or the environment can increase the risk of NPC. In contrast, several food items, including fresh vegetables, fruits, and green tea, have been reported to be inversely associated with NPC development [12,13]. Consumption of vegetables [14,15], as well as green tea [13,16], has been found to lower the risk of NPC. It has been suggested that the protective effect of vegetables and green tea may result from antioxidant ingredients. However, the underlying mechanisms have not been fully elucidated yet.

**Figure 1:**
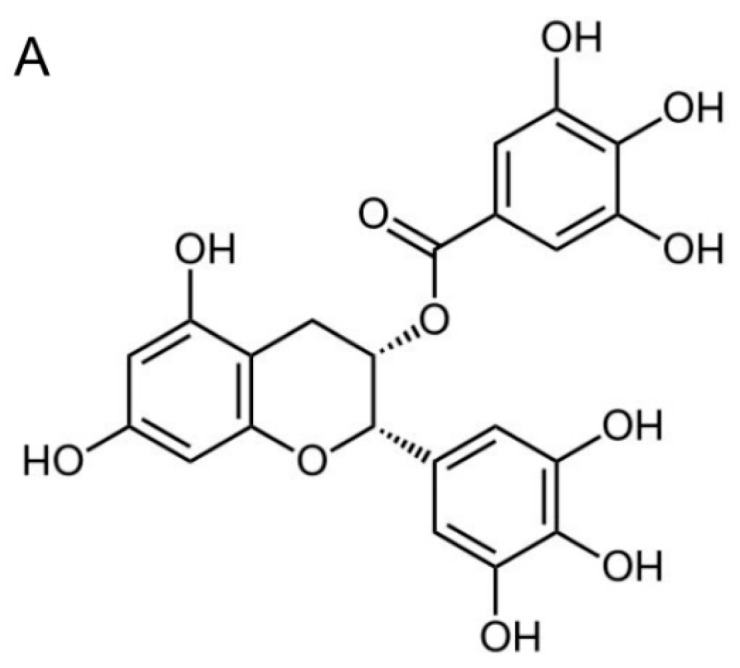

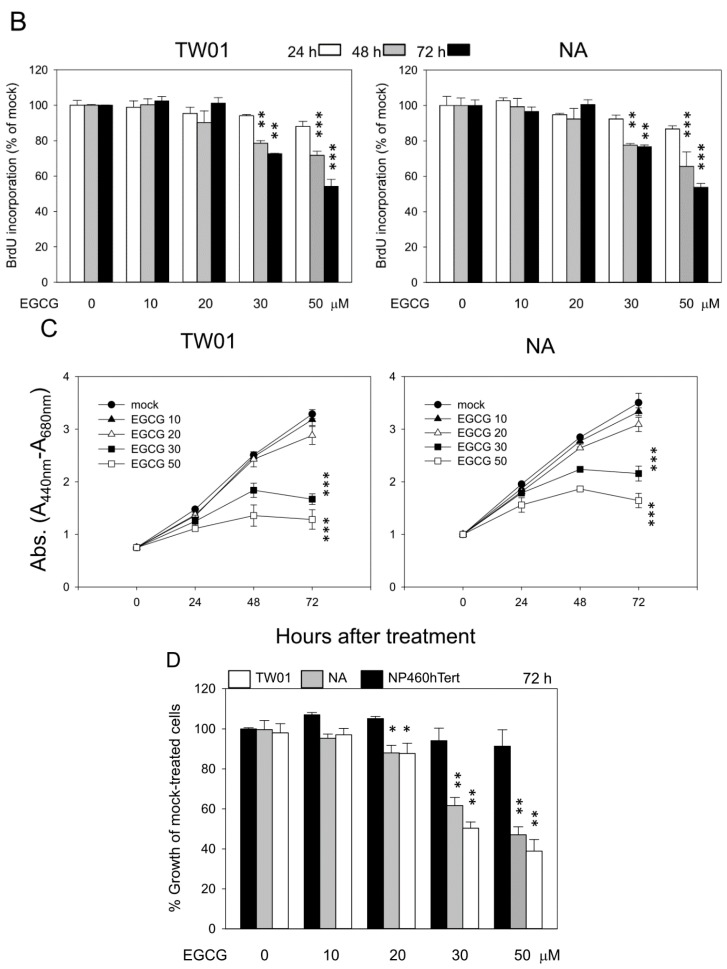
(−)-Epigallocatechin-3-gallate (EGCG) inhibits the proliferation of nasopharyngeal carcinoma (NPC) but not immortalized NP460hTert cells. (**A**) The chemical structure of EGCG; (**B**) The proliferation of TW01 and NA cells under EGCG treatment. Cells were treated with various concentrations of EGCG (0, 10, 20, 30 and 50 μM) for 24, 48 and 72 h. The proliferation of cells is presented as % BrdU incorporation of mock-treated cells. Data indicate the average value of triplicates (mean ± SD). **: *p* < 0.01; ***: *p* < 0.001, compared to mock-treated cells; (**C**) The viability assay of TW01 and NA cells under EGCG treatment. Cells were treated with various concentrations of EGCG for 24, 48 and 72 h. The viable cells were determined using a standard WST-1 assay. The X axis represents the absorbance value of (A_440 nm_ − A_680 nm_). Data indicate the average value of triplicates (mean ± SD). ***: *p* < 0.001, compared to mock-treated cells; (**D**) TW01, NA and NP460hTert cells were exposed to various concentrations of EGCG for 72 h. The viability of cells is presented as % growth of mock-treated cells. Data indicate the average value of triplicates (mean ± SD). *: *p* < 0.05; **: *p* < 0.01, compared to the NP460hTert cells.

Tea (*Camellia sinensis*) is widely consumed for its characteristic flavor and potential health benefits. Epidemiologic studies have demonstrated that consumption of green tea reduces the risk of cancers, including breast, lung, stomach, colon, liver, and pancreatic cancers [17,18,19,20]. A typical cup of green tea contains 100–150 mg of tea polyphenols, which are also known as catechins [21]. Several studies have shown that tea polyphenols inhibit the growth of cancer cells [22]. The major green tea polyphenol is (−)-epigallocatechin-3-gallate (EGCG, Figure 1A), which comprises more than 50% of total tea polyphenols [21]. EGCG has been found to possess profound chemopreventive and antitumor activities, which have been reported to result from inhibition of several signal transduction pathways related to carcinogenesis [18,23,24].

Although EGCG has been proved to be effective in inhibiting several types of cancer cells, its effect on NPC cells has not been well demonstrated. Previous studies have indicated that EGCG induced growth arrest, apoptosis [25,26], and inhibit stem-like characteristics in NPC cell lines [27]. Because of the limited availability of appropriate cell lines, most NPC studies were conducted on an EBV-negative cell background. This is because most NPC cell lines have lost their EBV genome during isolation and culture [28,29]. Although transfection of such cells with individual EBV latent genes, such as latent membrane protein 1 (LMP1), may provide a model for study, such cells do not reflect the authentic circumstances of NPC *in vivo*, which is positive for EBV infection. We have generated EBV-positive NPC cell lines in a previous study and have established xenografted mouse models of NPC using these cells [30,31]. The EBV-positive NPC cell lines were found to have enhanced malignancies as compared to EBV-negative cells [31]. In this study, an EBV-negative TW01 cell and an EBV-positive NA cell, which represents the two typical types of NPC *in vivo*, were used to study the effect of EGCG on NPC cells. Experiments *in vitro* indicated that EGCG inhibits the proliferation of NPC cells but does not affect the growth of an immortalized, non-malignant nasopharyngeal cell. Treatment with EGCG also reduced the migration, invasion, and spheroid formation in NPC cells. Following inoculation of NA cells into severe combined immunodeficiency (SCID) mice to generate an NPC tumor model, oral administration of EGCG effectively inhibited the proliferation of the tumors. Subsequent investigations revealed that the up-regulation of cell adhesion molecules, suppression of matrix metalloproteinases (MMP)-2 and MMP-9, and induction of apoptosis via activation of the caspase pathway were involved in the EGCG-induced inhibition. Our results provide evidence that EGCG may be potent as a chemopreventive or adjuvant agent for treatment of NPC.

## 2. Results

### 2.1. (−)-Epigallocatechin-3-gallate (EGCG) Inhibits the Proliferation of Nasopharyngeal Carcinoma (NPC) Cells but not Immortalized Nasopharyngeal Epithelial Cells

A BrdU incorporation assay was performed to determine the proliferation of cells under EGCG treatment (Figure 1B). At 10 and 20 μM of EGCG treatment, no difference in cell proliferation was observed, regardless of treatment periods (24, 48 and 72 h). At 24 h of 30 and 50 μM EGCG treatment, a slight reduction of proliferation was observed in both TW01 and NA cells (reduction < 10%, Figure 1B). As the treatment time was increased, the anti-proliferative effect of EGCG became more prominent. Compared to the mock-treated cells, the proliferation of both cells treated with 30 or 50 μM EGCG was significantly reduced at 48 and 72 h. This result indicates that EGCG can reduce the proliferation of NPC cells in a time- and dose-dependent manner. To further elucidate the effect of EGCG treatment, a cell viability assay was carried out to determine the cytotoxicity of EGCG on NPC cells. When compared to mock-treated cells, treatment with EGCG at 10 and 20 μM did not have significant effect on the cell viability at 24 and 48 h. Only after 72 h of 20 μM EGCG treatment was a slight reduction of viable cell numbers observed in TW01 and NA cells (Figure 1C). When the treatment doses were increased to 30 and 50 μM of EGCG, the cytotoxic effect of EGCG became more marked. Compared to the mock-treated cells, the viability of both TW01 and NA cells treated with 30 or 50 μM EGCG was significantly reduced at 48 and 72 h (Figure 1C). The viability of NPC cells at 72 h was lower than that after 48 h of treatment with 30 or 50 μM EGCG, indicating that EGCG may induce cell death with prolonged treatment. Suppression of proliferation by EGCG was found to be more marked in the EBV-negative TW01 cells, as compared to the EBV-positive NA cells at 48 and 72 h of treatments. Because EGCG has been shown to inhibit specifically the proliferation of cancer cells but not their normal counterparts, we compared the effect of EGCG on these two NPC cells and a telomerase-immortalized, non-malignant human nasopharyngeal epithelial (NP) cell line, NP460hTert [32]. Interestingly, after 72 h of treatment, EGCG did not show adverse effect on NP460hTert cells, regardless of the concentration (Figure 1D). Only a minor, but insignificant, reduction of cell proliferation was observed after treatment of NP460hTert cells with 30 or 50 μM EGCG. In contrast, the inhibitory effect of EGCG was very prominent on two NPC cells. Compared to NP460hTert cells, the reduction of two NPC cell growth was significant with 20 μM EGCG (*p* < 0.05), and greater with 30 or 50 μM EGCG (*p* < 0.01) treatment (Figure 1D). Taken together, these data indicate that EGCG can inhibit the proliferation of NPC cells in a time- and dose-dependent manner, while not affecting the growth of non-malignant NP cells.

### 2.2. EGCG Inhibits Nasopharyngeal Carcinoma (NPC) Cell Migration and Invasion

**Figure 2:**
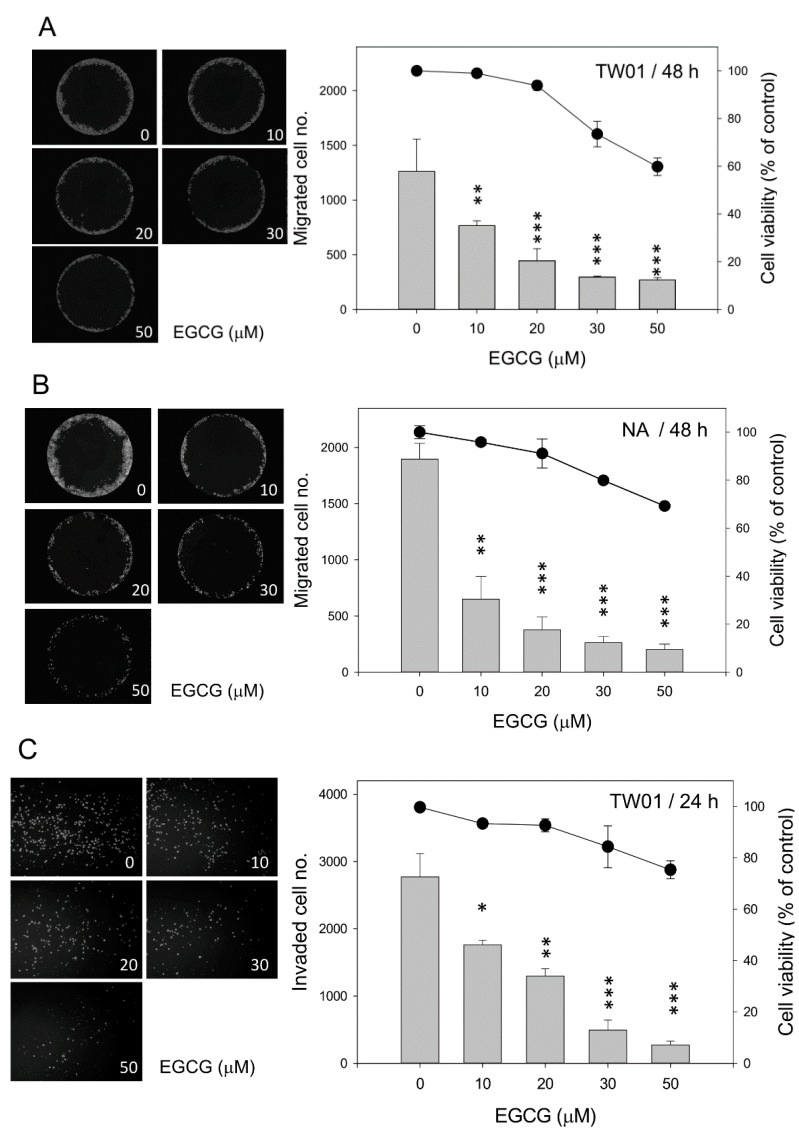

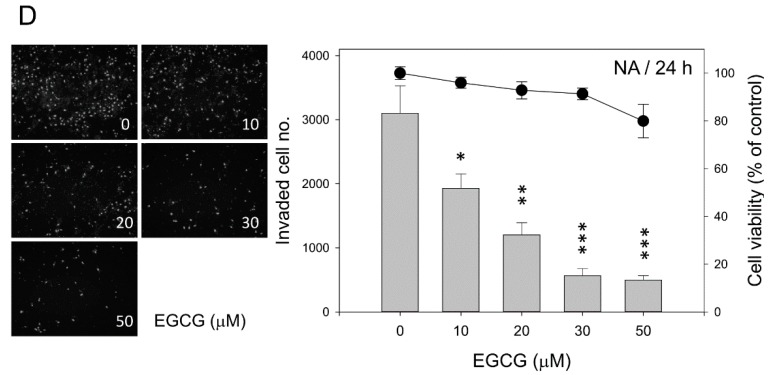
EGCG inhibits the migration and invasiveness of NPC cells. Cells were treated with various concentrations of EGCG for the times indicated. (**A**,**B**) The migration of TW01 and NA cells was determined by the number of cells that had migrated into the central blank area at 48 h (**left panel**). The bar plots represent the number of migrated cells and the line and dot plot indicates the viability of cells (**right panel**, the viability data were derived from the data of Figure 1C by defining the mock group as 100% at 48 h). Data indicate the average value of triplicates (mean ± SD). **: *p* < 0.01; ***: *p* < 0.001, compared to the mock-treated cells; (**C**,**D**) The invasiveness of TW01 and NA cells was determined by the number of cells that had invaded and transmigrated to the lower surface of the transwell membrane at 24 h (**left panel**). The bar plots represent the number of invading cells and the line and dot plot indicates the viability of cells (**right panel**, the viability data were derived from the data of Figure 1C by defining the mock group as 100% at 24 h). Data indicate the average value of triplicates (mean ± SD). *: *p* < 0.05; **: *p* < 0.01; ***: *p* < 0.001, compared to the mock-treated cells.

Cell migration and invasion assays were carried out to determine the preventive effects of EGCG on NPC cells. EGCG treatment had a profound inhibitory effect on cell migration and invasion, even at a low dose (Figure 2). When examined at 48 h, the migration of both TW01 and NA cells was markedly reduced after EGCG treatment at all concentrations tested (Figure 2A,B). With 10 μM EGCG treatment, there was a 40% and 65% drop in migration of TW01 and NA cells, respectively, while the cell growth was not affected. It seems that the inhibitory effect of EGCG was very remarkable for the highly-motile NA cells, as compared to the TW01 cells with lower migration ability. Although cell proliferation was suppressed at higher doses of EGCG, the reduction of migration apparently exceeded the extent of growth inhibition. A similar result was observed in the invasion assay at 24 h. While the cell growth was not affected, there was an approximate 35% reduction in invasiveness with 10 μM EGCG treatment in both cells (Figure 2C,D). Treatment with a higher concentration of EGCG reduced the invasiveness of both NPC cells further, while the proliferation of the cells was slightly inhibited. These data indicate that EGCG can inhibit NPC cell migration and invasion effectively, even at a concentration when cell growth was not affected.

### 2.3. EGCG Up-Regulates the Expression of Cell Adhesion Molecules E-Cadherin and β-Catenin

**Figure 3:**
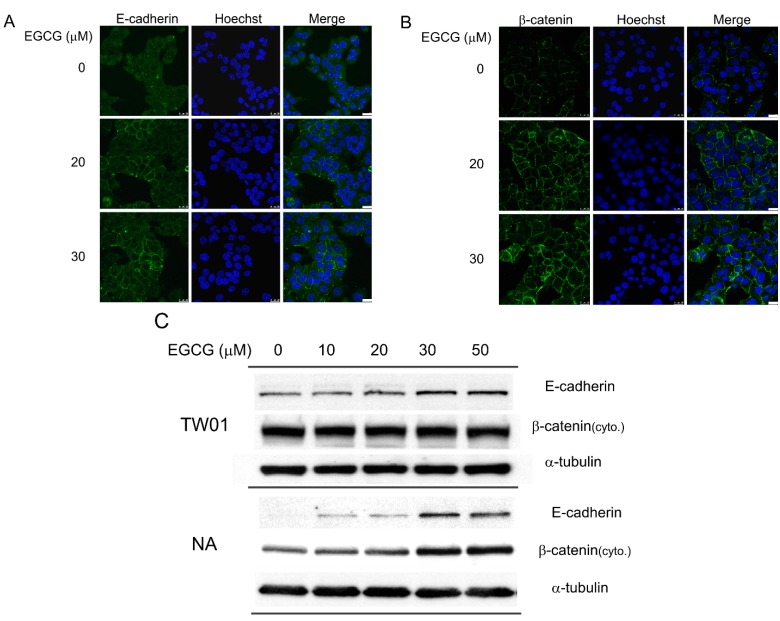
EGCG up-regulates the expression of cell adhesion molecules. NPC cells were treated with various concentrations of EGCG for 24 h. (**A**) The expression of E-cadherin was revealed by confocal microscopy in NA cells. Cell nuclei were stained with Hoechst 33258. The white bar at the lower corner represents a length of 25 μm; (**B**) The expression of β-catenin was determined by confocal microscopy in NA cells. Cell nuclei were stained with Hoechst 33258. The white bar at the lower corner represents a length of 25 μm; (**C**) Western blot analysis of E-cadherin and cytoplasmic β-catenin expression at 24 h in NPC cells. α-tubulin is detected as a loading control.

We observed a marked reduction in cell migration and invasion after EGCG treatment. To clarify whether changes in the expression of cell adhesion molecules were involved in the inhibition of migration and invasion, we investigated the expression of E-cadherin and β-catenin in these cells. Treatment with EGCG for 24 h induced the expression of E-cadherin, which assembled at the cell-cell junctions of NA cells (Figure 3A). The E-cadherin protein was almost undetectable in mock-treated NA cells (Figure 3A). Following treatment with EGCG at increasing concentrations, the expression of E-cadherin was up-regulated markedly. Treatment with EGCG for 24 h also increased the intensity of β-catenin staining at the cell-cell junctions in NA cells (Figure 3B). The amount of E-cadherin and cytoplasmic β-catenin protein also was revealed by immunoblotting after EGCG treatment (Figure 3C). In TW01 cells, the expression of E-cadherin was up-regulated as the dose of EGCG increased, while the expression of cytoplasmic β-catenin remained constant, perhaps due to the yet high amount expression of this protein. For mock-treated NA cells, the expression of E-cadherin was almost undetectable; however, as the treatment of EGCG increased, E-cadherin was up-regulated significantly. In contrast to TW01 cells, NA cells revealed a dose-dependent increase of cytoplasmic β-catenin expression as the dose of EGCG increased. These data indicate that EGCG can up-regulate the expression of the adhesion molecules E-cadherin and β-catenin, which may be responsible for the reduction of cell migration and invasion observed in NPC cells (Figure 2).

### 2.4. EGCG Suppressed the Activity of Matrix Metalloproteinase 2 (MMP-2) and MMP-9, Supposedly through Regulating the Extracellular Signal-Regulated Kinase (ERK) Signaling Pathways

**Figure 4:**
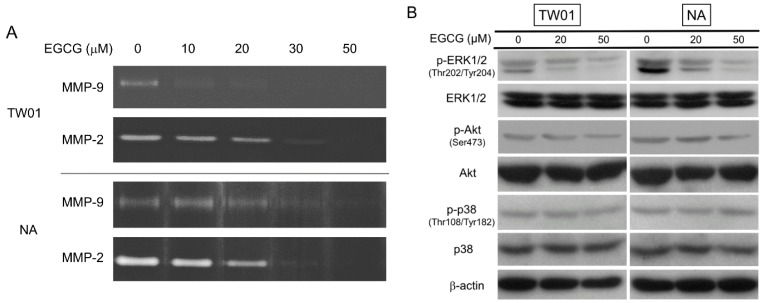

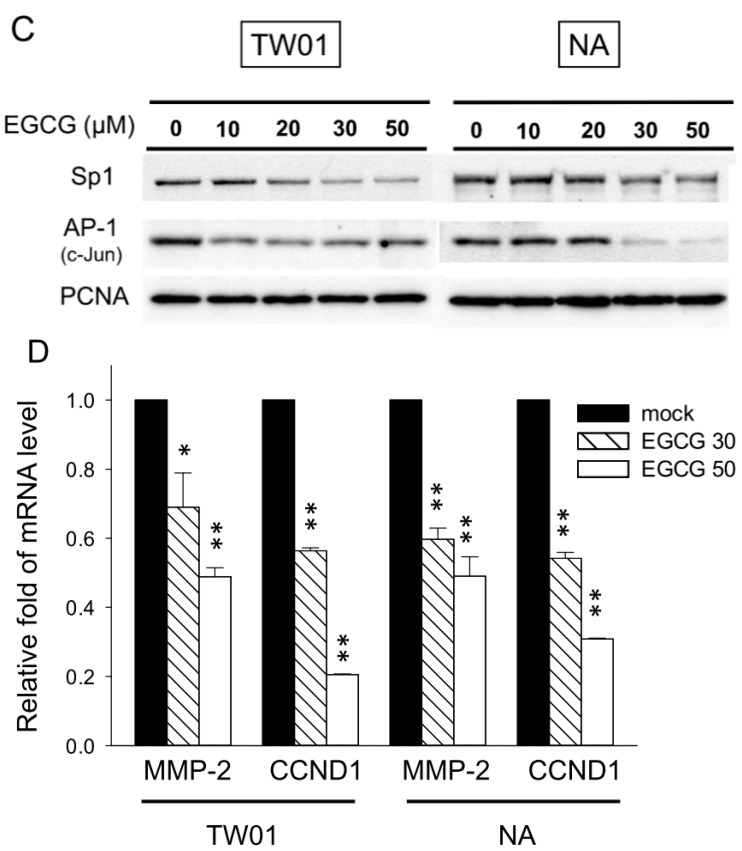
EGCG suppresses MMP-2 and MMP-9 activity through regulating the ERK signaling pathways. NPC cells were treated with various concentrations of EGCG. (**A**) Gelatin zymography of MMP-2 and MMP-9 activity in NPC cell supernatants after EGCG treatment for 24 h; (**B**) Western blot analysis of ERK1/2, phospho-ERK1/2 (Thr202/Tyr204), Akt, phospho-Akt (Ser473), p38, and phospho-p38 (Thr108/Tyr182) at 9 h of EGCG treatment in NPC cells. β-actin is detected as a loading control; (**C**) Western blot analysis of nuclear AP-1 (c-Jun) and Sp1 at 12 h of EGCG treatment in NPC cells. PCNA (proliferating cell nuclear antigen) is detected as a loading control; and (**D**) qRT-PCR of MMP-2 and CCND1 in NPC cells. The expression level of MMP-2 and CCND1 in mock-treated cells was adjusted as the base line (1.0-fold) and the relative expression level of genes in EGCG-treated cells was determined accordingly. Data indicate the mean expression level ± SD. *: *p* < 0.05; **: *p* < 0.01, compared to the mock-treated cells.

In addition to adhesion molecules, matrix metalloproteinases (MMPs) are capable of degrading extracellular matrix (ECM) proteins and play a major role in determining cell behavior, such as migration/invasion, differentiation, angiogenesis, and host defense. Among these MMPs, the activity of two gelatinases, MMP-2 and MMP-9, was found to be particularly associated with tumor metastasis [33]. Because treatment with EGCG can inhibit migration and invasion of NPC cell (Figure 2), the activity of MMP-2 and MMP-9 was determined by gelatin zymography. The EBV-positive NA cells had a higher MMP-2 and MMP-9 enzyme activity, as compared to the EBV-negative TW01 cells under untreated condition (Figure 4A). This may reflect the fact that NA cells have slightly higher invasion ability than TW01 cells in the invasion assay (Figure 2C,D). Upon treatment, the MMP-2 activity was significantly reduced in the supernatant of TW01 and NA cells as the dose of EGCG increased. MMP-9 activity also decreased notably, though its activity was weaker than MMP-2 in these two cells. The expression of MMP-2 and MMP-9 is reported to be regulated by several signaling pathways, including the mitogen activated protein kinase (MAPK) and phosphatidylinositol 3-kinase/Akt (PI3K/Akt) signaling pathways, which in turns affect the downstream transcription factor AP-1 and Sp1 that controls the promoter activity of MMP-2 and MMP-9 [34]. We therefore investigated the effect of EGCG on these signaling factors in the NPC cells. Treatment of EGCG reduced the phosphorylation of extracellular signal-regulated kinase (ERK) in TW01 and NA cells, while the total form of this protein remained relatively constant (Figure 4B). In contrast, the inhibition of Akt phosphorylation was less prominent and the level of p38 was not changed under EGCG treatment. Additionally, the downstream nuclear levels of AP-1 and Sp1 were also decreased in TW01 and NA cells as the phosphorylation of ERK reduced by increasing EGCG treatment (Figure 4C). A quantitative reverse transcription PCR (qRT-PCR) assay was performed to confirm the effect of nuclear AP-1 and Sp1 reduction by EGCG. MMP-2 and cyclin D1 (CCND1) are two genes in which their promoter activity is modulated by AP-1 and Sp1 [34,35]. By comparing to mock-treated cells, the mRNA levels of MMP-2 and CCND1 were significantly decreased in EGCG-treated cells (Figure 4D). This result indicated that reduced nuclear translocation of AP-1 and Sp1 by EGCG may decrease the expression of MMP-2 and CCND1. Altogether, these results may indicate that EGCG can reduce the gelatinases activity of NPC cells through suppression of ERK phosphorylation and inhibition of AP-1/Sp1 transactivation.

### 2.5. EGCG Reduces Spheroid Formation by NPC Cells in Culture

**Figure 5:**
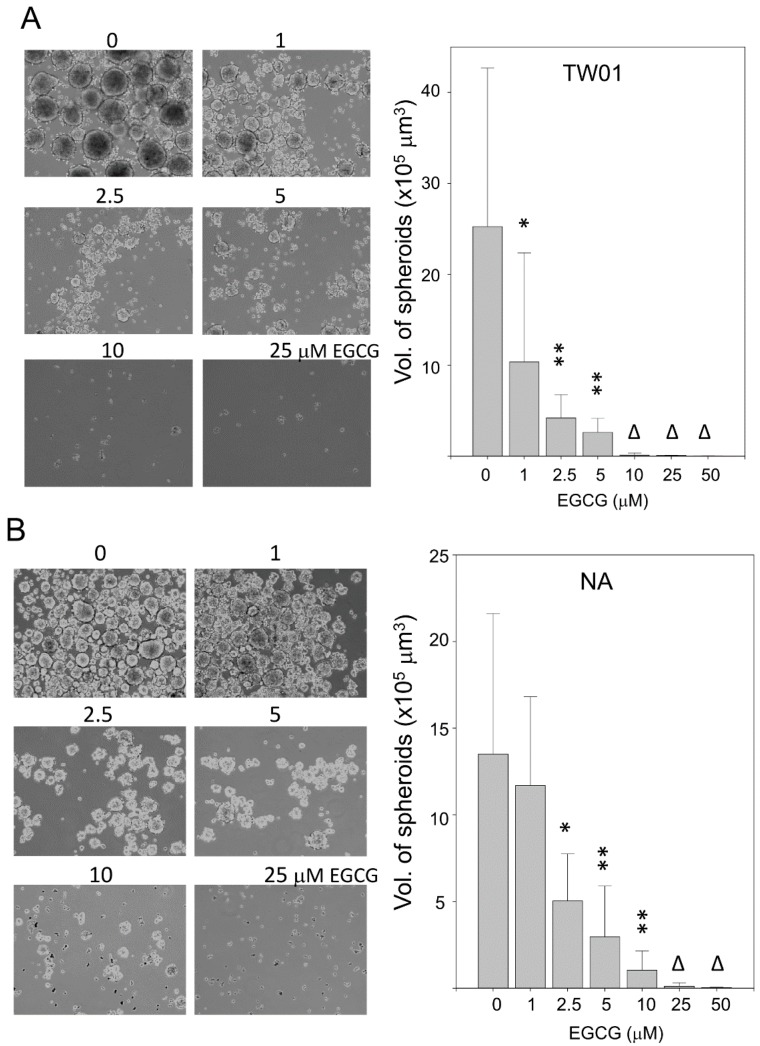
EGCG reduces the spheroid formation of NPC cells in culture. Cells were cultured in single-cell suspension for 7 days with EGCG treatment. The volumes of the spheroids were measured and are presented as a bar chart. Data indicate the average volume of spheroids (mean ± SD). At least 100 spheroids were measured for each experiment. *: *p* < 0.05; **: *p* < 0.01, compared to the mock-treated cells. Δ: No solid spheroid formation was observed. (**A**) TW01 cells; and (**B**) NA cells.

Tumor spheroids are multicellular tumor masses formed in suspension culture and are indicative in many biological studies [36]. Here, the inhibitory effect of EGCG on spheroid formation by NPC cells was evaluated. Single cell suspensions of cells were treated with various doses of EGCG and incubated for 7 days for spheroid formation. Under mock-treated condition, the two NPC cells were capable of forming large spheroids at suspension culture (Figure 5). The spheroids of TW01 cells were found to be larger than that of NA cells (average 2.5 × 10^6^ and 1.8 × 10^6^ μm^3^, respectively). For TW01 cells, treatment with 1.0 μM of EGCG induced a dramatic 60% reduction of spheroid volume, compared to the mock-treated cells (Figure 5A). As the concentration of EGCG increased, the formation of spheroids was further inhibited in a dose-dependent manner. At concentrations greater than 10 μM EGCG, there was no spheroid formation observed in the suspension culture of TW01 cells. For NA cells, the 1.0 μM of EGCG treatment did not lead to a significant decrease of spheroid volume (Figure 5B). As the concentration of EGCG was increased to 2.5 μM, the volume of spheroids was reduced to 37%, compared to mock-treated cells. The formation of NA spheroids was also significantly inhibited by EGCG in a dose-dependent manner. At concentrations greater than 25 μM EGCG, there was no spheroid formation observed in the suspension culture. These data revealed that EGCG has a very outstanding inhibitory effect on spheroid formation by NPC cells, and the EBV-negative TW01 cells are more susceptible to EGCG inhibition than the EBV-positive NA cells.

### 2.6. EGCG Inhibits NPC Tumor Growth in Vivo

**Figure 6:**
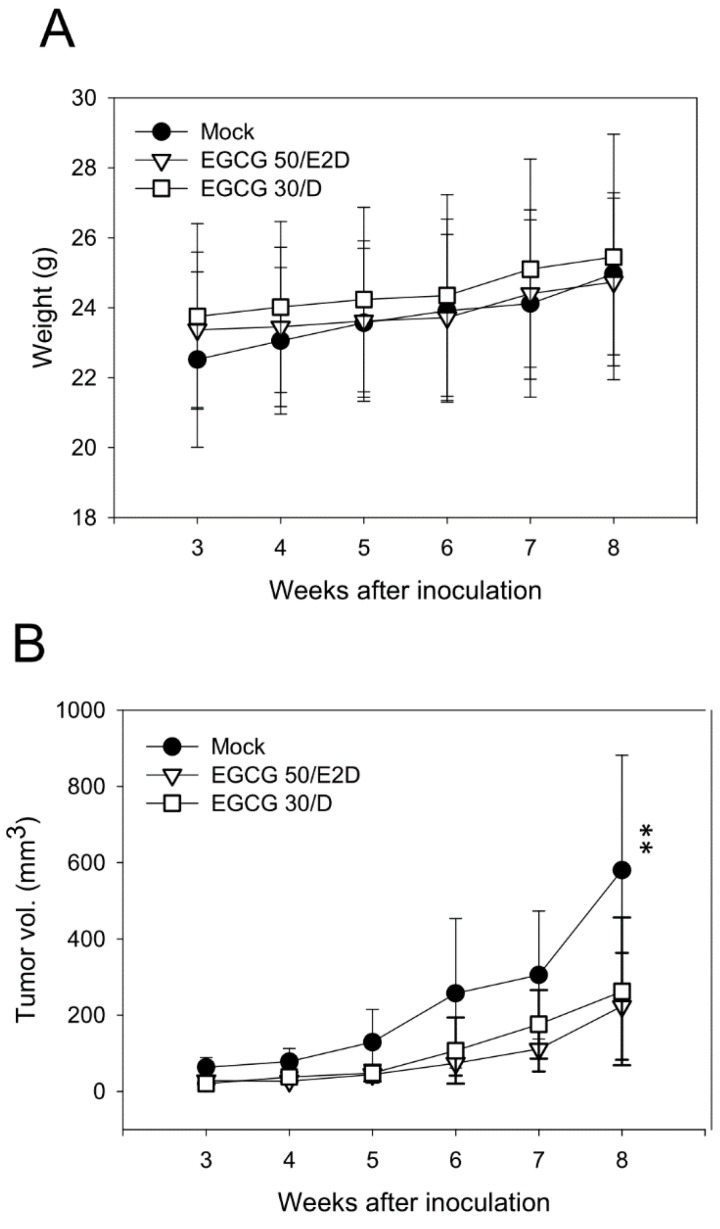

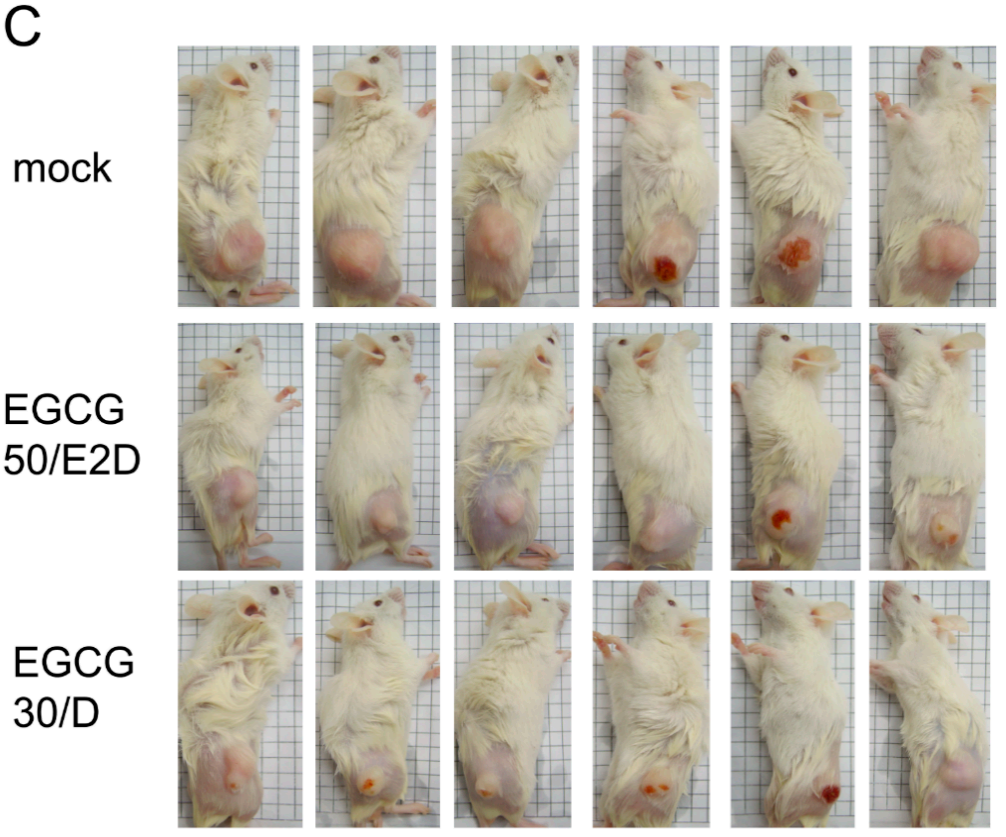
EGCG inhibits NPC tumor growth *in vivo*. NA cells were inoculated subcutaneously into SCID mice. Two groups of mice received EGCG administration: one with 50 mg/kg of EGCG every 2 days (the “50/E2D” group) and the other with 30 mg/kg of EGCG every day (the “30/D” group). The mice of the mock-treated group received only water. (**A**) The record of animal body weight during the experiment (*n* = 6 mice for each group); (**B**) The tumorigenicity of NA cells was evaluated by the size of the tumors at the inoculation site using a standard procedure at 7-day intervals post-injection. Data indicate mean tumor volume (*n* = 6) ± SD. **: *p* < 0.01, compared to the EGCG-treated groups; and (**C**) Representative photographs of tumor nodules in the mice at the 8th week.

To evaluate the effect of EGCG on tumor growth, a tumorigenesis assay was performed using SCID mice inoculated with NPC xenografts and monitored periodically by tumor volume. We chose NA cells in this study since previous study had shown that NA cells have a superior ability of tumor formation than TW01 cells in mice [31]. Two groups of mice were administered EGCG by oral gavage. One group of mice received a dose of 50 mg/kg EGCG every 2 days (50/E2D), while the other group received a dose of 30 mg/kg EGCG every day (30/D). Mock-treated (water only) mice constituted the control group. Figure 6A shows the effects of oral administration of EGCG on the body weights of the mice. During the study, the body weights of all three groups increased gradually without significant loss or variation. This indicates that treatment with EGCG did not have noticeable adverse effects on the animals during the experiment. Moreover, the volume of tumor nodules was significantly reduced in mice administered EGCG, compared to the mock-treated group (Figure 6B,C). Eight weeks after inoculation, the tumor volume of the mock group was three times as large as those of the EGCG-treated groups. Although the treatment program differed between the two EGCG-treated groups (50/E2D and 30/D), no significant difference in tumor sizes was observed. Taken together, these results indicate that EGCG can inhibit NPC tumor growth *in vivo* without an apparent adverse effect on the treated animals.

### 2.7. EGCG Up-Regulates the Expression of p53/p21 and Induces Apoptosis of NA Cells via Caspase 3 Activation

**Figure 7:**
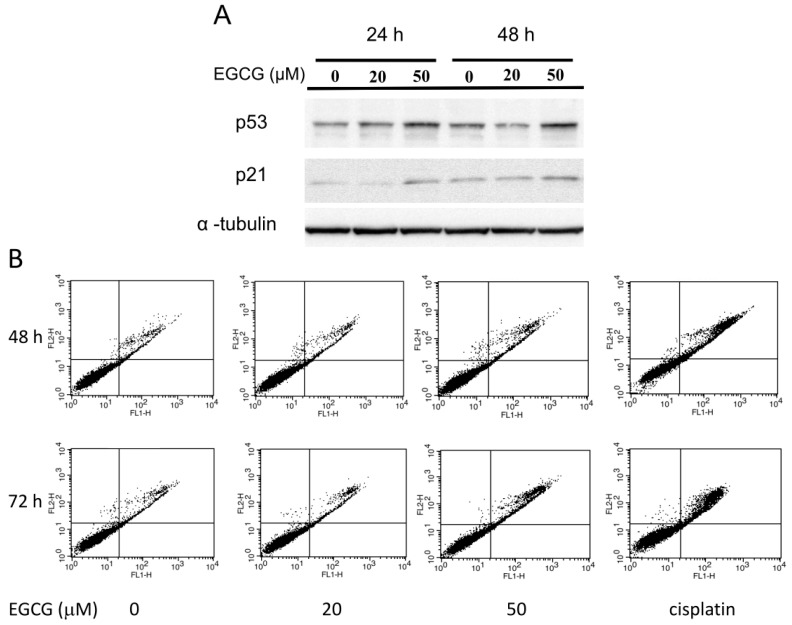

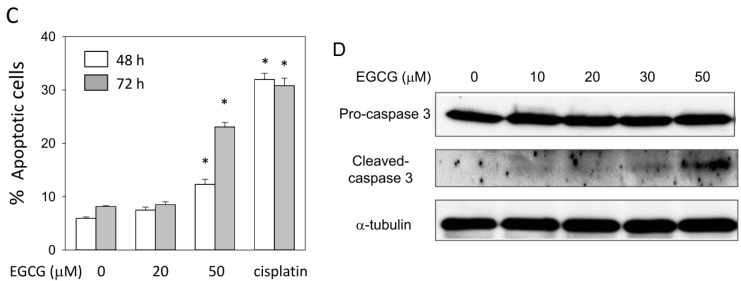
EGCG up-regulates the expression of p53 and p21, and induces apoptosis of NPC cells via caspase 3 activation. NA cells were treated with various concentrations of EGCG for the times indicated. (**A**) Western blot analysis of p53 and p21 in NA cells at 24 and 48 h. α-tubulin is detected as a loading control; (**B**) The apoptosis of NA cells was quantified using flow cytometry after staining with annexin-V and PI. Cisplatin-treated (5 μM) cells were included as a positive control for apoptosis. Representative scatter plots of annexin-V (*x*-axis) and PI (*y*-axis) were shown. Each dot on the plot represents a count event in cytometry analysis; (**C**) The percentage of apoptotic cells was summed up by the percentage of stained cells in the upper and lower right panels of the scatter plot. Data indicate the value of mean ± SD. *: *p* < 0.05, compared to the mock-treated cells; (**D**) Western blot analysis of cleaved caspase 3 after 24 h of EGCG treatment. α-Tubulin is detected as a loading control.

The tumorigenesis assay showed that administration of EGCG can reduce the NPC xenografts growth *in vivo*. This result implied that treatment with EGCG may inhibit NPC cell proliferation. Many studies have revealed that EGCG can inhibit cell cycle progression by various mechanisms [37,38,39]. In NA cells, treatment of EGCG was found to up-regulate the expression of p53 and p21 protein levels at 24 and 48 h (Figure 7A). The elevation of these two cell-cycle regulator proteins may in part contribute to the growth inhibition of NPC cells by EGCG. In addition, the cell viability assay had shown that treatment with EGCG can not only inhibit NPC cell proliferation but also reduce the viability of cells at 72 h (Figure 1C), which is an implication for induced cell death. An annexin-V/PI flow cytometry assay was performed to evaluate if apoptotsis is involved in reduction of NPC cells after EGCG treatment (Figure 7B). Treatment with 20 μM EGCG did not induce an increase in the apoptotic marker in NA cells, regardless of 48 or 72 h of treatment. In contrast, treatment with 50 μM EGCG induced marked apoptosis of NA cells at 48 and 72 h (Figure 7B,C). Compared to the mock-treated group, the apoptotic cells increased to two-fold at 48 h and to 3-fold at 72 h in 50 μM EGCG-treated cells. These results indicate that EGCG can induce apoptosis in NA cells. Caspases play a critical role in the process of apoptosis of various cells [40]. To explore the role of caspase in EGCG-induced apoptosis of NA cells, we examined the activation of caspase 3, a critical regulator of apoptotic pathways. After exposure to various concentrations of EGCG for 24 h, the level of cleaved caspase-3 was found to be increased in NA cells following treatment with 30 or 50 μM EGCG (Figure 7D). These results indicated that prolonged treatment with EGCG can induce growth arrest by up-regulation of p53/p21 and elicit apoptosis via the activation of caspase pathways in NA cells.

### 2.8. EGCG Suppresses the Nuclear Translocation of NF-κB and β-Catenin

**Figure 8:**
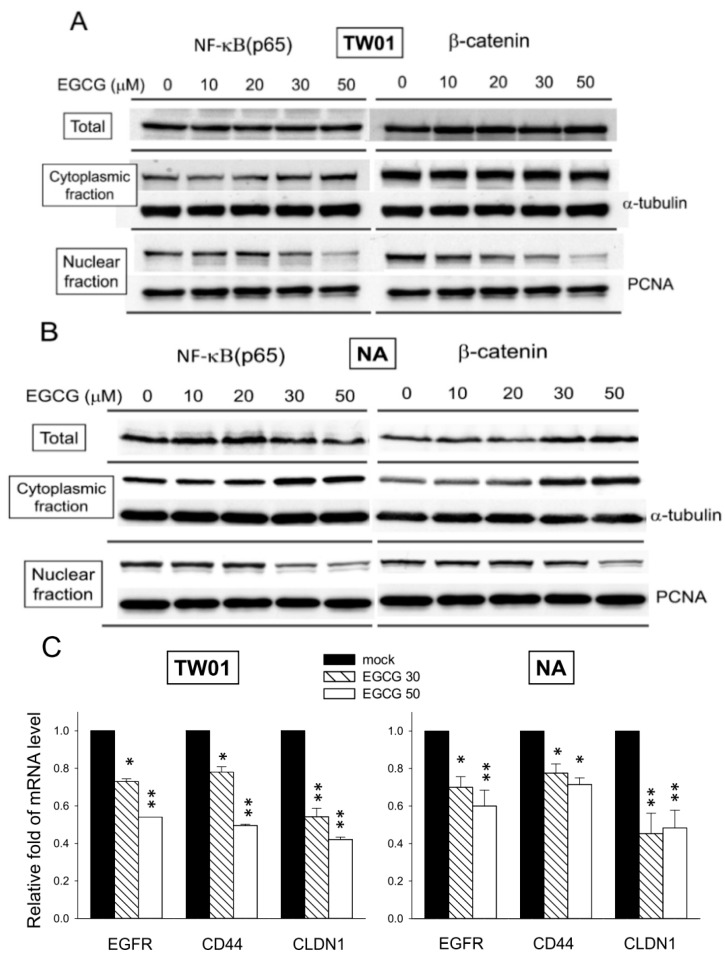
EGCG suppresses the nuclear translocation of NF-κB and β-catenin and the expression of downstream genes. Western blot analysis of total, cytoplasmic, and nuclear fractions of NPC cells after 24 h of EGCG treatment is presented. The levels of NF-κB and β-catenin are shown. α-Tubulin and PCNA are detected as a loading control for the cytoplasmic and nuclear fractions, respectively. (**A**) TW01 cells; (**B**) NA cells; and (**C**) qRT-PCR of EGFR, CD44 and CLDN1 in NPC cells. The RNA level of these genes in mock-treated cells was adjusted as the base line (1.0-fold) and the relative gene expression level in EGCG-treated cells was determined accordingly. Data indicate the mean expression level ± SD. *: *p* < 0.05; **: *p* < 0.01, compared to the mock-treated cells.

NF-κB, a key regulator that controls many cell programs, including proliferation and survival, is reported to be deregulated in many cancer cells [41]. NF-κB regulates several anti-apoptotic genes, such as TNF receptor-associated factor 1 (TRAF1) and TRAF2, which are vital in apoptotic process [42]. Here, exposure to EGCG for 24 h was found to increase the cytoplasmic level while decreasing the nuclear level of NF-κB in both TW01 and NA cells (Figure 8A,B). It seems that EGCG treatment increased the cytoplasmic retention while reducing the nuclear translocation of NF-κB. In the previous section, we have shown that EGCG treatment up-regulates the cytoplasmic level of β-catenin at cell–cell junctions (Figure 3B,C). In addition to cell adhesion, β-catenin is also an intracellular signal transducer in the Wnt signaling pathway that translocates into the nucleus and acts as a transcriptional regulator that controls many cellular functions including proliferation and migration [43]. The level of nuclear β-catenin was found to be reduced significantly in TW01 cells following EGCG treatment (Figure 8A). The level of nuclear β-catenin was also found to be decreased in NA cells after EGCG treatment, but the degree of reduction was more prominent at higher EGCG doses (>30 μM, Figure 8B). To confirm the effect of reduced nuclear levels of NF-κB and β-catenin on downstream gene expression after EGCG treatment, a qRT-PCR was performed to reveal the mRNA levels of epidermal growth factor receptor (EGFR), CD44, and claudin-1 (CLDN1). The expression of EGFR and CLDN1 are modulated by NF-κB and β-catenin, respectively [44,45]. CD44 is reported to be regulated both by NF-κB and β-catenin [46,47]. Comparing to mock-treated cells, the mRNA levels of EGFR, CD44 and CLDN1 were significantly decreased in EGCG-treated NPC cells (Figure 8C). While the decrease of these three mRNAs was prominent in TW01 cells, the reduction of EGFR and CD44, although significant, was less intense in EBV-positive NA cells after EGCG treatment. This result indicated that reduced nuclear level of NF-κB and β-catenin by EGCG may decrease the expression of EGFR, CD44, and CLDN1. These data showed that EGCG can suppress the nuclear translocation of NF-κB and β-catenin in NPC cells, thus lowering their transcriptional activity and may contribute to the inhibitory effects of EGCG.

## 3. Discussion

NPC is an endemic cancer that is prevalent particularly in southern China, Taiwan, Southeast Asia, and North Africa [1]. Genetic, environmental and microbial factors have been incriminated in the carcinogenesis of NPC [2,3]. Epidemiological studies have shown that several chemicals, including phorbols, butyrates and *N*-nitroso compounds, are associated with the development of NPC [6,7,8,9]. These chemicals have been found to present in herbal medicines and foodstuffs commonly consumed by local residents in NPC high risk area [6,10,15]. EBV infection is also implicated in the development of NPC [4]. It has been postulated that the interactions between chemicals and EBV may enhance the incidence of NPC [10,48], however the underlying mechanisms are not fully understood. Recently we showed that 12-*O*-tetradecanoylphorbol-13-acetate (TPA), sodium butyrate and *N*-methyl-*N*'-nitro-*N*-nitrosoguanidine (MNNG) can enhance synergistically the reactivation of EBV in NPC cells and lead to genome instability and alteration of gene expression, with resultant enhancement of NPC carcinogenesis [11,49,50]. These studies imply that certain chemical ingredients from foods may induce the reactivation of EBV and enhance the carcinogenesis of NPC. On the other hand, several foods, including vegetables, fruits and green tea, have been reported to have an inverse association with NPC [12,13,51]. Intake of fresh vegetables has been reported to be associated with a 36% reduction in the risk of NPC [12]. Recently, consumption of green tea also has been shown to lower the risk of NPC [13,16]. Green tea has been shown to have an inhibitory effect on many types of cancer cells [17,18,19,20]. The chemopreventive property of green tea has mainly been attributed to EGCG, which has potent antioxidant properties and is the major component of green tea polyphenols. In this study, we showed that EGCG can inhibit the proliferation, migration, invasive properties, and induce apoptosis, of both EBV-positive and -negative NPC cell lines. The tumorigenesis assay also showed that EGCG can effectively reduce tumor growth *in vivo*. These results indicate that EGCG may be a promising candidate for chemoprevention or adjuvant therapy of NPC.

Tea polyphenols have been shown to have growth inhibitory effects on cancer cells but do not have adverse effects on normal cells [22,52]. In this study, exposure of EGCG was found to suppress the proliferation of NPC cells but not immortalized NP460hTert cells (Figure 1D). It is suggested that the differences in the biochemical characteristics between cancer and normal cells may lead to this outcome [22]. The fact that EGCG was found to induce apoptosis in NPC cells (Figure 7B,C), while not affecting the growth of non-malignant NP460hTert (Figure 1D) with equivalent treatment, suggests that components in the cell-cycle regulation and apoptotic pathways could be the targets for EGCG in NPC cells. It has been reported that EGCG can inhibit tumor cell growth by inducing the expression of the cyclin-dependent kinase inhibitor p21 protein, and this effect is correlated with the increase in p53 levels [37]. We showed that EGCG treatment up-regulated the expression of cell cycle regulator p53 and p21, which could lead to the inhibition of cell proliferation in NPC cells (Figure 7A). Prolonged treatment with EGCG was also shown to induce a marked amount of apoptosis in NPC cells but had little effect on NP460hTert cells (Figure 7C and Figure 1D). Activation of caspases by EGCG had been demonstrated in cancer cells [53]. In this study, the EGCG-induced apoptosis of NPC cells was found to mediate through the activation of caspase 3 (Figure 7C,D). The specific effects of EGCG on the growth arrest and apoptosis induction of NPC cells should make it a safety and effective agent for chemoprevention or therapy of NPC.

Metastasis is the major cause of cancer mortality. The advancements in therapy of NPC have been improved substantially; however, patients with late stage disease frequently suffer from relapse or metastasis, the major cause of mortality. Migration and invasion by cancer cells are complicated processes involving alteration of cell adhesion molecules, so that cells can detach from each other or from the extracellular matrix (ECM), and expression of proteolytic enzymes that degrade the ECM and basal membrane. In this study, we have shown that EGCG can reduce the migration and invasiveness of NPC cells dramatically, even at a dose that did not show adverse effects on cell growth (Figure 2). Treatment with EGCG induced the expression of E-cadherin and up-regulated the amount of cytoplasmic β-catenin (Figure 3). An increase in the amount of E-cadherin and β-catenin at cell–cell junctions can immobilize the cells and lead to reduced motility of NPC cells. In addition, the activity of MMP-2 and MMP-9 was found to be suppressed significantly in NPC cells following EGCG treatment (Figure 4A). MMP-2 (gelatinase A) and MMP-9 (gelatinase B), which degrades type IV collagen, the major structural component of basement membranes, are overexpressed in various malignant tumors and are known to play crucial roles in tumor migration and invasion [33]. Expression of MMP-2 and MMP-9 has been reported to be associated with cervical lymph node metastasis in NPC [54]. It is apparent that up-regulation of adhesion molecules and suppression of MMP activities by EGCG can lead to reduction of the migration and invasiveness of NPC cells. The expression of MMP-2 and MMP-9 has been reported to be regulated by several transcriptional factors, including AP-1, Sp1, NF-κB and β-catenin [55,56]. Inhibition of MAPK and PI3K/Akt pathways had been shown to inhibit both AP-1 and Sp1-mediate MMP gene expressions [57,58]. In our study, treatment of EGCG reduced the phosphorylation of ERK and decreased the nuclear levels of AP-1 and Sp1 in NPC cells (Figure 4B,C). These results indicate that EGCG can reduce the gelatinases activity of NPC cells though suppression of ERK phosphorylation, which results in inhibition of AP-1 and Sp1-mediate MMP expression (Figure 4D).

Inhibition of nuclear translocation of NF-κB and β-catenin was observed in NPC cells after EGCG treatment (Figure 8A,B). With increasing concentration of EGCG, the expression level of total NF-κB was decreased. NF-κB was found to accumulate in the cytoplasm but the amount in the nucleus decreased. NF-κB has been proved to involve in cell cycle regulation and cell growth and survival [59]. Inhibition of NF-κB by EGCG has been shown to induce apoptosis of cancer cells via activation of caspases 3, 8, and 9 [53]. Therefore, the decrease of nuclear NF-κB may be involved in the growth arrest and the activation of capase 3, which resulted in the apoptosis of NPC cells (Figure 7 and Figure 8). It had been demonstrated previously in NPC cells that inhibition of NF-κB was mediated by EGCG through the inhibition of IκBα phosphorylation and degradation [25], which sequestered NF-κB in an inactive state in the cytoplasm. Although the total and cytoplasmic level of β-catenin was increased in NA cells, the nuclear level of β-catenin was found to decline after EGCG treatment, both in TW01 and NA cells (Figure 8A,B). Other than associating with E-cadherin as part of the adherens junctions, β-catenin also interacts with other proteins and acts as a transcriptional regulator when it translocates into the nucleus and is part of the Wnt/β-catenin signaling pathway [43]. An increased level of nuclear β-catenin has been associated with malignancies [60]. Recently, it has been shown that suppression of β-catenin expression by siRNA resulted in down-regulation of MMP-2 and MMP-9 in NPC cell lines [61]. Hence, the decrease of the nuclear level of β-catenin could also contribute to the reduction of MMP-2 and MMP-9 activity in NPC cells. It is not clear how EGCG treatment inhibit the translocation of β-catenin in NPC cells. Glycogen synthase kinase 3 (GSK3), a critical downstream element of the PI3k/Akt pathways, has been shown to phosphorylate β-catenin and target it for degradation [62]. In this study, the phosphorylation status of Ser9 in GSK3β was investigated to see if it is affected by EGCG in NPC cells. Since phosphorylation in the Ser9 of GSK3β reduces its kinase activity, we expected to see a decline of p-GSK3β after EGCG treatment. Instead, an increase of p-GSK3β (Ser9) was observed in NPC cells (Figure S1). This result may indicate that the decrease of nuclear level of β-catenin is not mediated by GSK3 phosphorylation, but by other mechanisms yet to be determined. The decreased mRNA levels of EGFR, CD44 and CLDN1 also implicated that reduced nuclear levels of NF-κB and β-catenin by EGCG may result in the down-regulation of the downstream genes (Figure 8C). Taken together, these results indicate that a decrease of nuclear NF-κB and β-catenin levels by EGCG treatment can be associated with the growth inhibition, down-regulation of MMP activity and induction of apoptosis of NPC cells.

Tumor spheroids are large, multicellular tumor masses formed *in vitro*, usually in suspension culture. The characteristics of tumor spheroid are considered to be closer to tumors *in vivo* than single-layer cells [63]. Recently, spheroid formation is considered to be a convenient way for enriching cancer stem-like cells [36]. Cells that capable of forming spheroids are considered to be self-renewal and can differentiate into multiple cell types [64]. A recent study by Lin *et al*., has shown that EGCG was less effective in growth inhibition and apoptosis induction of sphere-derived NPC cells than its parental cells, but was still efficient in inhibiting their stem-cell like characteristics, such as colony formation and invasiveness [27]. It seems that the sphere-derived NPC cells had acquired an increased resistance to EGCG-induced growth arrest. In difference to their study, our study revealed that EGCG is very effective in inhibition of single NPC cell from growing into spheroids in suspension culture. The inhibition was so remarkable that the effective concentration was much lower than those in the monolayer viability assay (Figure 1 and Figure 5). Our result may reflect that EGCG can efficiently inhibit the “stem-like” cells of NPC from self-renew and differentiating into multiple cells in a suspension culture. Nevertheless, even if the spheroid is formed, Lin’s study indicated that EGCG is still effective in suppressing the invasion and self-renewal capacity of NPC cells [27].

In this study, an EBV-negative TW01 cell and an EBV-positive NA cell, which represents the two typical types of NPC *in vivo*, were used for comparison the difference in response to EGCG treatment in NPC cells. NA cells, which harbor the EBV genome, exhibit several enhanced malignancies as compared to TW01 cells [31]. These differences are suggested to result from the expression of several viral oncogenes in NA cells. As compared with TW01 cells, NA cells were found to have a somewhat higher resistance to EGCG-induced inhibition in cell proliferation (Figure 1B,C), MMP9 expression (Figure 4A), spheroid formation (Figure 5), nuclear translocation of β-catenin (Figure 8A,B), and less downstream target gene reduction (Figure 8C). It is possible that expression of latent EBV genes had conferred the NA cells with certain degree of resistance, which requires further elucidation. In this study, in the range of 1 to 50 μM EGCG, the inhibition tendency of TW01 and NA cells were quite similar in most assays. The difference in dose response between TW01 and NA cells were within one or two orders (2×–4×). Furthermore, administration of EGCG was capable of inhibiting NA cell xenograft growth, which has an enhanced tumorigenisity as compared to TW01 cells in mice [31]. Although NA cells did reveal a slightly higher resistance to EGCG-induced inhibition, our data revealed that under most conditions, EGCG is effective in inhibiting both the EBV-negative and -positive NPC cells.

Administration of EGCG has been shown to be very well tolerated in human and animal models. Oral intake of EGCG is the most convenient means of administration and is relatively safe, even in considerably high doses [65]. We have shown that administration of EGCG can effectively reduce the tumor growth *in vivo* without adverse effects on animal body weight (Figure 6). Two administration programs, one with 50 mg/kg EGCG every 2 days and the other with 30 mg/kg EGCG every day, were applied to mice bearing NA tumors. The tumor volumes of these two groups were significantly lower than the mock-treated group and there was no difference between the two treatments. This may imply that the amount of EGCG received was sufficient for inhibition of NPC tumor growth, regardless of treatment intervals. The growth of tumors in mice was examined while they were sacrificed for tumor sample collection. In the mock-treated mice, the tumors were so large that they were found to invade deeply into the underneath thigh muscle tissues, with some of them reaching the femur (*n* = 6 of thigh muscle invasion, 3 reaching the femur, data not shown). In contrast, the tumors of the EGCG-treated groups were largely confined to the dermal region, with some of them showing an initial stage of shallow muscle invasion (*n* = 2 for the 50/E2D and *n* = 3 for the 30/D group, data not shown). This result may in part reflect that EGCG administration not only has an inhibitory effect on tumor growth, but also reduces the tissue invasiveness of NA cells *in vivo*.

It has been shown that EGCG also exerts its antitumor activity through inhibiting angiogenesis [66,67]. The development of new blood vessels is critical for tumorigenesis not only for nourishing growing tumor but also for metastasis [68]. It has been demonstrated that oral consumption of green tea by mice inhibited angiogenesis [66]. In our tumorigenesis assay, the EGCG-treated mice had significant reduced tumor sizes compared to the mock-treated group (Figure 6), and the tumors were largely confined to the dermal region as described above. This inhibition may partially be ascribed to the antiangiogenic effect of EGCG. EGCG had been shown to down-regulate the expression of vascular endothelial growth factor (VEGF), which is a potent angiogenic protein that has mitogenic and chemotactic effects on vascular endothelial cells [69]. Interestingly, ERK1/2 has been reported to be important signaling factors in the expression of VEGF [70]. In our study, we have found that EGCG suppresses ERK1/2 phosphorylation and the downstream AP-1 and Sp1 transactivation in NPC cells (Figure 4B,C). Although not evaluated in this study, it is possible that EGCG may exert its antiangiogenic activity by down-regulating the expression of VEGF via inhibiting the ERK pathways. In addition, EGFR is reported to be an upstream mediator of mitogenic factors VEGF and IL-8 [71]. In our study, the expression of EGFR was found to be suppressed after EGCG treatment in NPC cells (Figure 8C). All in all, these observations may suggest that the antitumor effect of EGCG is mediated in part by inhibition of angiogenesis.

## 4. Experimental Section

### 4.1. Cell Lines and Chemicals

NPC-TW01 (TW01) is an EBV-negative nasopharyngeal carcinoma cell line derived from the nasopharyngeal tumors of a Chinese patient [28]. NA cells, an EBV-positive NPC cell line that harbors the viral genome and mimics the *in vivo* condition of NPC cells, was derived from re-infection of the TW01 cell with EBV [30]. Cells were cultured in Dulbecco’s modified Eagle’s medium (DMEM) supplemented with 10% fetal bovine serum (HyClone, Waltham, MA, USA) at 37 °C with 5% CO_2_. G418 (400 μg/mL, Amresco, Solon, OH, USA) was added to the medium of NA cells to maintain the EBV genome in the cells [30]. The immortalized human nasopharyngeal (NP) cell line NP460hTert was cultured and maintained as described previously [32]. (−)-Epigallocatechin-3-gallate (EGCG; [(2*R*,3*R*)-5,7-Dihydroxy-2-(3,4,5-trihydroxyphenyl)chroman-3-yl] 3,4,5-trihydroxybenzoate; CAS no. 989-51-5), cisplatin (*cis*-diammineplatinum(II) dichloride, CAS no. 15663-27-1) and gelatin were obtained from Sigma–Aldrich (St. Louis, MO, USA). EGCG was dissolved in dimethyl sulfoxide (DMSO) as a stock solution of 100 mM and further diluted in culture medium to appropriate final concentration when used, with the final content of DMSO not exceeding 0.5%.

### 4.2. Proliferation and Cytotoxicity Assay

Cell proliferation assay was performed by the instruction of the Cell Proliferation ELISA BrdU kit (Roche Diagnostics, Mannheim, Germany). Cells were seeded in 96-well plates at a density of 1 × 10^4^ cells/100 μL per well and treated with EGCG at various concentrations for the times indicated. At the end of treatments, 10 μL of 5-bromo-2'-deoxyuridine (BrdU) was added to the culture medium and incubation continued at 37 °C for 2 h. The incorporated BrdU was detected by anti-BrdU-peroxidase and the formation of color substrate was measured using a microplate reader at an absorption wavelength of 370 nm.

Cell viability assay was performed using the WST-1 reagent (Roche Diagnostics) according to the protocols suggested by the manufacturer. Briefly, cells were seeded in 96-well plates at a density of 1 × 10^4^ cells/100 μL per well and treated with EGCG at various concentrations for the times indicated. At the end of treatments, 10 μL of 2-(4-iodophenyl)-3-(4-nitrophenyl)-5-(2,4-disulfophenyl)-2H-tetrazolium (WST-1) was added to the culture medium and incubation continued at 37 °C for 2 h. The formation of dark red formazan was measured using a microplate reader at an absorption wavelength of 440 nm. To minimize the difference in the culture conditions between NPC and NP460hTert cells, 10% fetal bovine serum was added to the medium of NP460hTert cells during this assay. (NP460hTert was originally cultured in serum-free medium).

### 4.3. Cell Migration Assay

Cell migration assays were performed using Oris™ Migration Assay kits (Platypus Technologies, Madison, WI, USA) according to the protocols suggested by the manufacturer. Briefly, 5 × 10^4^ cells were inoculated into each well of 96-well plates while the central stoppers were inserted. Six hours after cell attachment, EGCG was added at various concentrations. The central stopper was removed after 12 h of incubation to allow the cell to migrate into the central area. After 48 h, the cells were fixed and stained with 50 μg/mL propidium iodide (Sigma–Aldrich). The cells that had migrated into the central area were photographed under a fluorescence microscope and the cell number was calculated.

### 4.4. Cell Invasion Assay

*In vitro* invasion assays were performed using HTS FluoroBlok inserts (Falcon, Cambridge, MA, USA) as described previously [31]. Briefly, the transwell membranes were coated with Matrigel (Becton Dickinson, Franklin Lakes, NJ, USA). 1 × 10^5^ cells were seeded onto the Matrigel-coated membranes and the inserts were incubated in 24-well plates with various concentrations of EGCG for 24 h. After incubation, the membranes were fixed with methanol and stained with 50 μg/mL propidium iodide. The cells that had invaded and transmigrated to the lower surface of the polycarbonate membrane were photographed under a fluorescence microscope and the cell number was calculated using AIS software (Imagine Research, Toronto, ON, Canada).

### 4.5. Immunofluorescence Staining

For immunofluorescence assay, cells were seeded onto round glass slips at a density of 1 × 10^5^ cells/well in 24-well plate for 24 h before treatment. After EGCG treatment, cells were washed twice in phosphate buffered saline (PBS) and followed by fixation and permeabilization with ice-cold methanol for 15 min. The cells were incubated with primary antibody overnight at 4 °C, and then washed and incubated with secondary anti-IgG–fluorescein isothiocyanate (1:5000) for 1 h at room temperature. Cell nuclei were stained with Hoechst 33258 (1 μg/mL) for 10 min. Immunostained cells were washed thoroughly with PBS, mounted and examined under a confocal microscope. Antibodies against E-cadherin and β-catenin (Cell Signaling, Danvers, MA, USA) were used as the primary antibodies in these analyses.

### 4.6. Western Blot Assay

Cells were cultured and treated with EGCG as described. After treatment, cells were collected either as the total lysates or subjected to cytoplasmic and nuclear fractionation using an NE-PER extraction kit (Thermo Scientific, Brookfield, WI, USA). Lysates were separated in a 10% polyacrylamide gel and transferred onto a nitrocellulose membrane. The blot was then probed with primary and secondary antibodies using a standard procedure, as described previously [31]. The expression profile of the proteins was visualized using a Western Lightening-ECL kit (PerkinElmer, Waltham, MA, USA). Antibodies against p53, p21, ERK, Akt, p38, Sp1, AP-1 (c-Jun), pGSK3β, E-cadherin, β-catenin, NF-κB (p65), caspase-3, α-tubulin, β-actin and proliferating cell nuclear antigen (PCNA) (Cell Signaling) were used as the primary antibodies in these analyses.

### 4.7. Gelatin Zymography

MMP-2 and MMP-9 enzymatic activities in culture supernatants were determined by SDS-PAGE gelatin zymography. Gelatinases present in the culture supernatant degrade the gelatin matrix in gel, leaving a clear band after staining the gel for protein [72]. Briefly, 1 × 10^5^ cells were seeded in the wells of 24-well plates for 12 h. After incubation, the medium was replaced with serum-free DMEM containing various concentrations of EGCG for 24 h. After treatment, the supernatants, which contained the secreted MMPs, were collected and denatured in the absence of a reducing agent and electrophoresed in 7.5% SDS-PAGE containing 0.1% (*w*/*v*) gelatin. The gels were then incubated in the presence of 2.5% Triton X-100 at room temperature for 2 h and subsequently at 37 °C overnight in a reaction buffer (10 mM CaCl_2_, 0.15 M NaCl, and 50 mM Tris, pH 7.5). Thereafter, the gels were stained with 0.25% Coomassie Blue and proteolysis was detected as a white band against a blue background.

### 4.8. Spheriod Formation Assay

NPC cells grown as monolayers were detached to generate a single-cell suspension. The cell suspension was diluted and transferred to 10 cm non-treated plates with 5 × 10^4^ cells in 10 mL medium with 4% FBS. The plates were incubated under standard cell culture conditions at 37 °C, 5% CO_2_ in humidified incubators. EGCG was added at various concentrations after 24 h of incubation. Spheroids were collected by brief centrifugation on the 7th day of EGCG treatment. The volumes of the spheroids were calculated under the microscope from their equatorial (*a*) and polar (*b*) diameters using the formula volume = *a*^2^*b* × 4/3π.

### 4.9. Tumor Growth in Severe Combined Immunodeficiency Mice

Six-week-old SCID (severe combined immunodeficiency) mice were used for this study. Mice were divided into three groups of six and kept under sterile conditions. The protocols were approved by the Institutional Animal Care and Use Committee of National Health Research Institutes, Taiwan (IACUC-098089-A, approved 31 December 2009). NA cells (1 × 10^6^ cells in each inoculation site) were suspended in serum-free DMEM and injected subcutaneously into the dorsal flanks of SCID mice. Administration of EGCG to the animals began 6 days after tumor inoculation to allow the time for establishment of tumors. Two groups of mice received EGCG administration by oral gavage. One group of mice received 50 mg/kg of EGCG dissolved in 100 μL water every 2 days (the “50/E2D” group), while the other group received 30 mg/kg of EGCG every day (the “30/D” group). The mice of mock-treated group received only water. Mice were examined weekly and tumor volumes were estimated from their length (*l*) and width (*w*), as measured by calipers, using the formula, tumor volume = *l w*^2^ × 0.52. Mice were sacrificed when the tumor volume of mock group reached approximately 1000 mm^3^.

### 4.10. Apoptosis Assay by Annexin-V and Propidium Iodide Staining

EGCG induced apoptosis was analyzed by flow cytometry with annexin-V Apoptosis Detection kit FITC (eBioscience, Affymetrix, San Diego, CA, USA) according to the procedure suggested by the manufacturer. Cells were cultured in 6-well plates at a density of 2 × 10^5^ cells per well and treated with EGCG as described. After treatment, the cells were detached and stained with annexin-V-FITC and propidium iodide (PI) labeling solution. Annexin-V and PI-positive cells were analyzed by flow cytometry (BD Biosciences, San Jose, CA, USA) and identified as apoptotic cells.

### 4.11. RNA Extraction and Semi-Quantitative Real-Time PCR

RNA was extracted from NPC cells using the RNAzol reagent (Sigma–Aldrich). For quantification of genes of interest, the RNA samples were reverse transcribed into cDNA using the RevertAid First Strand cDNA Synthesis kit (Thermo Scientific). One twentieth of the cDNA was used in semi-quantitative real-time PCR (qRT-PCR) for genes of interest using SensiFAST SYBR kit (Bioline, Taunton, MA, USA). The calculations for determining the level of gene expression were made using the cycle threshold (*C*_t_) method. Utilizing the human TBP (TATA box binding protein) gene as the internal control [73], the relative quantitation values of a target template for each sample were expressed as 2^−ΔΔ*C*t^, where ΔΔ*C*_t_ = Δ*C*_t_^treated^ − Δ*C*_t_^mock^. The primer sequences used in this study were designed by QuantPrime [74] and are listed in Table S1.

### 4.12. Statistical Analysis

Differences between multiple groups were analyzed by one-way ANOVA with Tukey’s method for pairwise comparisons. The *t*-test was used for comparisons of two groups. *p* < 0.05 was considered to be statistically significant.

## 5. Conclusions

We demonstrated that EGCG have selective growth inhibition on NPC cells but not on non-malignant NP cells. EGCG can inhibit proliferation, migration, invasion, spheroid formation, and induce apoptosis of NPC cells in culture and reduce tumor growth *in vivo*. The inhibition effect of EGCG is just about equally effective in both EBV-positive and -negative NPC cells. These results indicate that EGCG may serve as a potential candidate for chemoprevention or adjuvant therapy of NPC.

## Supplementary Materials

Supplementary materials can be found at http://www.mdpi.com/1422-0067/16/02/2530/s1.

## References

[1] De-The G., Roizman B. (1982). Epidemiology of Epstein-Barr virus and associated diseases in man. The Herpesviruses.

[2] Hildesheim A., Levine P.H. (1993). Etiology of nasopharyngeal carcinoma: A review. Epidemiol. Rev..

[3] McDermott A.L., Dutt S.N., Watkinson J.C. (2001). The aetiology of nasopharyngeal carcinoma. Clin. Otolaryngol. Allied Sci..

[4] Young L.S., Rickinson A.B. (2004). Epstein-Barr virus: 40 years on. Nat. Rev. Cancer.

[5] Ho J.H. (1972). Nasopharyngeal carcinoma (NPC). Adv. Cancer Res..

[6] Zou X.N., Lu S.H., Liu B. (1994). Volatile *N*-nitrosamines and their precursors in Chinese salted fish—A possible etological factor for NPC in china. Int. J. Cancer.

[7] Zur Hausen H., O’Neill F.J., Freese U.K., Hecker E. (1978). Persisting oncogenic herpesvirus induced by the tumour promotor TPA. Nature.

[8] Luka J., Kallin B., Klein G. (1979). Induction of the Epstein-Barr virus (EBV) cycle in latently infected cells by *n*-butyrate. Virology.

[9] Rickinson A.B., Kieff E., Knipe D.M., Howley P.M. (2001). Epstein-Barr Virus. Fields’ Virology.

[10] Hirayama T., Ito Y. (1981). A new view of the etiology of nasopharyngeal carcinoma. Prev. Med..

[11] Fang C.Y., Huang S.Y., Wu C.C., Hsu H.Y., Chou S.P., Tsai C.H., Chang Y., Takada K., Chen J.Y. (2012). The synergistic effect of chemical carcinogens enhances Epstein-Barr virus reactivation and tumor progression of nasopharyngeal carcinoma cells. PLoS One.

[12] Gallicchio L., Matanoski G., Tao X.G., Chen L., Lam T.K., Boyd K., Robinson K.A., Balick L., Mickelson S., Caulfield L.E. (2006). Adulthood consumption of preserved and nonpreserved vegetables and the risk of nasopharyngeal carcinoma: A systematic review. Int. J. Cancer.

[13] Hsu W.L., Pan W.H., Chien Y.C., Yu K.J., Cheng Y.J., Chen J.Y., Liu M.Y., Hsu M.M., Lou P.J., Chen I.H. (2012). Lowered risk of nasopharyngeal carcinoma and intake of plant vitamin, fresh fish, green tea and coffee: A case-control study in Taiwan. PLoS One.

[14] Zheng Y.M., Tuppin P., Hubert A., Jeannel D., Pan Y.J., Zeng Y., de The G. (1994). Environmental and dietary risk factors for nasopharyngeal carcinoma: A case-control study in Zangwu County, Guangxi, China. Br. J. Cancer.

[15] Feng B.J., Jalbout M., Ayoub W.B., Khyatti M., Dahmoul S., Ayad M., Maachi F., Bedadra W., Abdoun M., Mesli S. (2007). Dietary risk factors for nasopharyngeal carcinoma in Maghrebian countries. Int. J. Cancer.

[16] Ruan H.L., Xu F.H., Liu W.S., Feng Q.S., Chen L.Z., Zeng Y.X., Jia W.H. (2010). Alcohol and tea consumption in relation to the risk of nasopharyngeal carcinoma in Guangdong, China. Front. Med. China.

[17] Katiyar S.K., Mukhtar H. (1996). Tea consumption and cancer. World Rev. Nutr. Diet..

[18] Yang C.S., Maliakal P., Meng X. (2002). Inhibition of carcinogenesis by tea. Annual Rev. Pharmacol. Toxicol..

[19] Khan N., Mukhtar H. (2008). Multitargeted therapy of cancer by green tea polyphenols. Cancer Lett..

[20] Ji B.T., Chow W.H., Hsing A.W., McLaughlin J.K., Dai Q., Gao Y.T., Blot W.J., Fraumeni J.F. (1997). Green tea consumption and the risk of pancreatic and colorectal cancers. Int. J. Cancer.

[21] Yang C.S., Wang Z.Y. (1993). Tea and cancer. J. Natl. Cancer Inst..

[22] Chen Z.P., Schell J.B., Ho C.T., Chen K.Y. (1998). Green tea epigallocatechin gallate shows a pronounced growth inhibitory effect on cancerous cells but not on their normal counterparts. Cancer Lett..

[23] Brusselmans K., de Schrijver E., Heyns W., Verhoeven G., Swinnen J.V. (2003). Epigallocatechin-3-gallate is a potent natural inhibitor of fatty acid synthase in intact cells and selectively induces apoptosis in prostate cancer cells. Int. J. Cancer.

[24] Lambert J.D., Yang C.S. (2003). Mechanisms of cancer prevention by tea constituents. J. Nutr..

[25] Zhao Y., Yang L.F., Ye M., Gu H.H., Cao Y. (2004). Induction of apoptosis by epigallocatechin-3-gallate via mitochondrial signal transduction pathway. Prev. Med..

[26] Zhao Y., Tao Y.-G., Luo F.-J., Tang F.-Q., Tang M., Cao Y. (2004). Interference effect of epigallocatechin-3-gallate on targets of nuclear factor κB signal transduction pathways activated by EB virus encoded latent membrane protein 1. Int. J. Biochem. Cell Biol..

[27] Lin C.H., Shen Y.A., Hung P.H., Yu Y.B., Chen Y.J. (2012). Epigallocathechin gallate, polyphenol present in green tea, inhibits stem-like characteristics and epithelial-mesenchymal transition in nasopharyngeal cancer cell lines. BMC Complement. Altern. Med..

[28] Lin C.T., Wong C.I., Chan W.Y., Tzung K.W., Ho J.K., Hsu M.M., Chuang S.M. (1990). Establishment and characterization of two nasopharyngeal carcinoma cell lines. Lab. Investig..

[29] Glaser R., Zhang H.Y., Yao K.T., Zhu H.C., Wang F.X., Li G.Y., Wen D.S., Li Y.P. (1989). Two epithelial tumor cell lines (HNE-1 and HONE-1) latently infected with Epstein-Barr virus that were derived from nasopharyngeal carcinomas. Proc. Natl. Acad. Sci. USA.

[30] Chang Y., Tung C.H., Huang Y.T., Lu J., Chen J.Y., Tsai C.H. (1999). Requirement for cell-to-cell contact in Epstein-Barr virus infection of nasopharyngeal carcinoma cells and keratinocytes. J. Virol..

[31] Fang C.Y., Lee C.H., Wu C.C., Chang Y.T., Yu S.L., Chou S.P., Huang P.T., Chen C.L., Hou J.W., Chang Y. (2009). Recurrent chemical reactivations of EBV promotes genome instability and enhances tumor progression of nasopharyngeal carcinoma cells. Int. J. Cancer.

[32] Li H.M., Man C., Jin Y., Deng W., Yip Y.L., Feng H.C., Cheung Y.C., Lo K.W., Meltzer P.S., Wu Z.G. (2006). Molecular and cytogenetic changes involved in the immortalization of nasopharyngeal epithelial cells by telomerase. Int. J. Cancer.

[33] Liotta L.A., Tryggvason K., Garbisa S., Hart I., Foltz C.M., Shafie S. (1980). Metastatic potential correlates with enzymatic degradation of basement membrane collagen. Nature.

[34] Wang L., Zhang Z.G., Zhang R.L., Gregg S.R., Hozeska-Solgot A., LeTourneau Y., Wang Y., Chopp M. (2006). Matrix metalloproteinase 2 (MMP2) and MMP9 secreted by erythropoietin-activated endothelial cells promote neural progenitor cell migration. J. Neurosci..

[35] Klein E.A., Assoian R.K. (2008). Transcriptional regulation of the *cyclin D1* gene at a glance. J. Cell Sci..

[36] Hirschhaeuser F., Menne H., Dittfeld C., West J., Mueller-Klieser W., Kunz-Schughart L.A. (2010). Multicellular tumor spheroids: An underestimated tool is catching up again. J. Biotechnol..

[37] Liang Y.C., Lin-Shiau S.Y., Chen C.F., Lin J.K. (1999). Inhibition of cyclin-dependent kinases 2 and 4 activities as well as induction of Cdk inhibitors p21 and p27 during growth arrest of human breast carcinoma cells by (−)-epigallocatechin-3-gallate. J. Cell Biochem..

[38] Gupta S., Ahmad N., Nieminen A.L., Mukhtar H. (2000). Growth inhibition, cell-cycle dysregulation, and induction of apoptosis by green tea constituent (−)-epigallocatechin-3-gallate in androgen-sensitive and androgen-insensitive human prostate carcinoma cells. Toxicol. Appl. Pharmacol..

[39] Gupta S., Hussain T., Mukhtar H. (2003). Molecular pathway for (−)-epigallocatechin-3-gallate-induced cell cycle arrest and apoptosis of human prostate carcinoma cells. Arch. Biochem. Biophys..

[40] Earnshaw W.C., Martins L.M., Kaufmann S.H. (1999). Mammalian caspases: Structure, activation, substrates, and functions during apoptosis. Annu. Rev. Biochem..

[41] Dolcet X., Llobet D., Pallares J., Matias-Guiu X. (2005). NF-κB in development and progression of human cancer. Virchows Arch..

[42] Wang C.Y., Mayo M.W., Korneluk R.G., Goeddel D.V., Baldwin A.S. (1998). NF-κB antiapoptosis: Induction of TRAF1 and TRAF2 and c-IAP1 and c-IAP2 to suppress caspase-8 activation. Science.

[43] Kolligs F.T., Bommer G., Goke B. (2002). Wnt/β-catenin/Tcf signaling: A critical pathway in gastrointestinal tumorigenesis. Digestion.

[44] Nishi H., Neta G., Nishi K.H., Akers L.M., Rikiyama T., Proctor K.N., Murphy B.A., Johnson A.C. (2003). Analysis of the epidermal growth factor receptor promoter: The effect of nuclear factor-κB. Int. J. Mol. Med..

[45] Miwa N., Furuse M., Tsukita S., Niikawa N., Nakamura Y., Furukawa Y. (2001). Involvement of claudin-1 in the β-catenin/Tcf signaling pathway and its frequent up-regulation in human colorectal cancers. Oncol. Res..

[46] Hinz M., Lemke P., Anagnostopoulos I., Hacker C., Krappmann D., Mathas S., Dorken B., Zenke M., Stein H., Scheidereit C. (2002). Nuclear factor κB-dependent gene expression profiling of Hodgkin’s disease tumor cells, pathogenetic significance, and link to constitutive signal transducer and activator of transcription 5a activity. J. Exp. Med..

[47] Wielenga V.J., Smits R., Korinek V., Smit L., Kielman M., Fodde R., Clevers H., Pals S.T. (1999). Expression of CD44 in Apc and Tcf mutant mice implies regulation by the WNT pathway. Am. J. Pathol..

[48] Ito Y., Kawanishi M., Harayama T., Takabayashi S. (1981). Combined effect of the extracts from *Croton tiglium*, *Euphorbia lathyris* or *Euphorbia tirucalli* and *n*-butyrate on Epstein-Barr virus expression in human lymphoblastoid P3HR-1 and Raji cells. Cancer Lett..

[49] Huang S.Y., Fang C.Y., Tsai C.H., Chang Y., Takada K., Hsu T.Y., Chen J.Y. (2010). *N*-Methyl-*N*'-nitro-*N*-nitrosoguanidine induces and cooperates with 12-*O*-tetradecanoylphorbol-1,3-acetate/sodium butyrate to enhance Epstein-Barr virus reactivation and genome instability in nasopharyngeal carcinoma cells. Chem. Biol. Interact..

[50] Huang S.Y., Fang C.Y., Wu C.C., Tsai C.H., Lin S.F., Chen J.Y. (2013). Reactive oxygen species mediate Epstein-Barr virus reactivation by *N*-methyl-*N*'-nitro-*N*-nitrosoguanidine. PLoS One.

[51] Farrow D.C., Vaughan T.L., Berwick M., Lynch C.F., Swanson G.M., Lyon J.L. (1998). Diet and nasopharyngeal cancer in a low-risk population. Int. J. Cancer.

[52] Ahmad N., Feyes D.K., Nieminen A.L., Agarwal R., Mukhtar H. (1997). Green tea constituent epigallocatechin-3-gallate and induction of apoptosis and cell cycle arrest in human carcinoma cells. J. Natl. Cancer Inst..

[53] Gupta S., Hastak K., Afaq F., Ahmad N., Mukhtar H. (2004). Essential role of caspases in epigallocatechin-3-gallate-mediated inhibition of nuclear factor κB and induction of apoptosis. Oncogene.

[54] Zhang X., Guo Y., Ye Q., Yang Z., Dong Z. (1999). Study of the relation between MMP2, MMP9 and nasopharyngeal carcinoma. J. Clin. Otorhinolaryngol..

[55] Takahra T., Smart D.E., Oakley F., Mann D.A. (2004). Induction of myofibroblast MMP-9 transcription in three-dimensional collagen I gel cultures: regulation by NF-κB, AP-1 and Sp1. Int. J. Biochem. Cell Biol..

[56] Yan C., Boyd D.D. (2007). Regulation of matrix metalloproteinase gene expression. J. Cell Physiol..

[57] Byun H.J., Hong I.K., Kim E., Jin Y.J., Jeoung D.I., Hahn J.H., Kim Y.M., Park S.H., Lee H. (2006). A splice variant of CD99 increases motility and MMP-9 expression of human breast cancer cells through the AKT-, ERK-, and JNK-dependent AP-1 activation signaling pathways. J. Biol. Chem..

[58] Kuo L., Chang H.C., Leu T.H., Maa M.C., Hung W.C. (2006). Src oncogene activates MMP-2 expression via the ERK/Sp1 pathway. J. Cell Physiol..

[59] Joyce D., Albanese C., Steer J., Fu M., Bouzahzah B., Pestell R.G. (2001). NF-κB and cell-cycle regulation: The cyclin connection. Cytokine Growth Factor Rev..

[60] Korinek V., Barker N., Morin P.J., van Wichen D., de Weger R., Kinzler K.W., Vogelstein B., Clevers H. (1997). Constitutive transcriptional activation by a β-catenin-Tcf complex in APC^−/−^ colon carcinoma. Science.

[61] Song Y., Yang Q.X., Zhang F., Meng F., Li H., Dong Y., Han A. (2012). Suppression of nasopharyngeal carcinoma cell by targeting β-catenin signaling pathway. Cancer Epidemiol..

[62] Mills C.N., Nowsheen S., Bonner J.A., Yang E.S. (2011). Emerging roles of glycogen synthase kinase 3 in the treatment of brain tumors. Front. Mol. Neurosci..

[63] Yuhas J.M., Li A.P., Martinez A.O., Ladman A.J. (1977). A simplified method for production and growth of multicellular tumor spheroids. Cancer Res..

[64] Nicolis S.K. (2007). Cancer stem cells and “stemness” genes in neuro-oncology. Neurobiol. Dis..

[65] Isbrucker R.A., Edwards J.A., Wolz E., Davidovich A., Bausch J. (2006). Safety studies on epigallocatechin gallate (EGCG) preparations. Part 2: Dermal, acute and short-term toxicity studies. Food Chem. Toxicol..

[66] Cao Y., Cao R. (1999). Angiogenesis inhibited by drinking tea. Nature.

[67] Jung Y.D., Ellis L.M. (2001). Inhibition of tumour invasion and angiogenesis by epigallocatechin gallate (EGCG), a major component of green tea. Int. J. Exp. Pathol..

[68] Fidler I.J., Ellis L.M. (1994). The implications of angiogenesis for the biology and therapy of cancer metastasis. Cell.

[69] Plate K.H., Breier G., Weich H.A., Risau W. (1992). Vascular endothelial growth factor is a potential tumour angiogenesis factor in human gliomas *in vivo*. Nature.

[70] Jung Y.D., Nakano K., Liu W., Gallick G.E., Ellis L.M. (1999). Extracellular signal-regulated kinase activation is required for up-regulation of vascular endothelial growth factor by serum starvation in human colon carcinoma cells. Cancer Res..

[71] Bancroft C.C., Chen Z., Yeh J., Sunwoo J.B., Yeh N.T., Jackson S., Jackson C., van Waes C. (2002). Effects of pharmacologic antagonists of epidermal growth factor receptor, PI3K and MEK signal kinases on NF-κB and AP-1 activation and IL-8 and VEGF expression in human head and neck squamous cell carcinoma lines. Int. J. Cancer.

[72] Lengyel E., Gum R., Juarez J., Clayman G., Seiki M., Sato H., Boyd D. (1995). Induction of *M*_r_ 92,000 type IV collagenase expression in a squamous cell carcinoma cell line by fibroblasts. Cancer Res..

[73] Bieche I., Laurendeau I., Tozlu S., Olivi M., Vidaud D., Lidereau R., Vidaud M. (1999). Quantitation of MYC gene expression in sporadic breast tumors with a real-time reverse transcription-PCR assay. Cancer Res..

[74] Arvidsson S., Kwasniewski M., Riano-Pachon D.M., Mueller-Roeber B. (2008). QuantPrime—A flexible tool for reliable high-throughput primer design for quantitative PCR. BMC Bioinform..

